# Continuous Spike-Wave during Slow Wave Sleep and Related Conditions

**DOI:** 10.1155/2014/619079

**Published:** 2014-01-30

**Authors:** Nilika Shah Singhal, Joseph E. Sullivan

**Affiliations:** Department of Neurology, University of California, P.O. Box 0114, 505 Parnassus Avenue, M-798, San Francisco, CA 94143-0114, USA

## Abstract

Continuous spike and wave during slow wave sleep (CSWS) is an epileptic encephalopathy that presents with neurocognitive regression and clinical seizures, and that demonstrates an electroencephalogram (EEG) pattern of electrical status epilepticus during sleep, as defined by the Commission on Classification and Terminology of the International League Against Epilepsy 1989. CSWS is an age-related condition, typically presenting in children around 5 years of age, with clinical seizures which progress within 2 years to a severe epileptic encephalopathy. The pathophysiology of CSWS is not completely understood, but the corticothalamic neuronal network involved in sleep patterns is thought to be involved. Genetic predisposition and injury in early development are thought to play etiological roles. Treatment strategies have involved traditional anticonvulsants, hormonal therapies, and other newer techniques. Outcomes are fair, and the thought is that earlier diagnosis and intervention preserve neurocognitive development, as in the case of other epileptic encephalopathies. Further understanding of the mechanisms of CSWS may lead to improved therapeutic options and thus outcomes of children with CSWS.

## 1. Introduction

Electrical status epilepticus during sleep was first described in 1971 as EEG abnormalities that became continuous with sleep onset and ceased to be continuous upon arousal, occupying at least 85% of slow sleep tracing [1]. These children did not demonstrate overt clinical manifestations; thus, the condition was described as “subclinical” or “electrical.” In this initial study, the authors described this condition as a form of encephalopathy given cognitive impairment in all children [1], and as more patients were described with this condition, it became clear that neurocognitive deterioration occurred at the same time as the electrical status epilepticus during slow wave sleep developed [2]. Subsequently, the International League Against Epilepsy (ILAE) opted to term the condition “continuous spike and waves during sleep” (CSWS) as a specific epilepsy syndrome characterized by continuous diffuse spike-waves occurring during slow-wave sleep, seen after the onset of seizures, that has a potentially poor outcome despite a noncatastrophic onset of seizures, related to the development of neurocognitive deficits (Commission on Classification and Terminology of the International League Against Epilepsy, 1989).

Electrical status epilepticus during sleep (ESES) is now the term typically used to describe solely the electrographic findings, while the CSWS syndrome remains a clinical diagnosis of the constellation of neurocognitive deterioration from baseline, affecting motor, language, cognitive, or behavioral development, and a significant proportion of non-REM sleep disrupted by spike-wave discharges [2].

## 2. Epidemiology

Continuous spike and waves during sleep (CSWS) is an age-related condition uniquely affecting children. While data are sparse, it is thought to represent 0.5% to 0.6% of all childhood epilepsy cases seen at tertiary referral epilepsy centers [3, 4]. There seems to be slight male predominance, about 60% to 40% [5–8].

## 3. Clinical Features

The classical presentation is a child with around 5 years of age, typically between age 4 and 7 years, who presents with new-onset seizures accompanied by mild developmental regression. While rarely presenting in children beyond age 10–12 years [5, 9], cases as young as two years of age have been reported [6, 7]. As many as 62–74% of children have been reported to have normal neurocognitive and motor function prior to onset of ESES [4, 10].

In up to 80% of children, seizures are the presenting symptom and typically occur out of sleep [2]. Children may present with focal clonic, primary and secondarily generalized tonic-clonic, and absence seizures [2], though as many as 80% of children present with only one seizure type [5]. Seizure onset tends to be initially benign and occur daily or more frequently in less than 20% of cases [5]. Once the ESES pattern appears, lateralizing features of seizures become less prominent, and absence and atonic seizures may occur [2]. The majority of cases, up to 70%, will have several seizures per day once the ESES pattern appears [5, 9, 11, 12].

Neurocognitive regression along with the development of ESES on EEG tends to appear within 2-3 years after seizure onset, typically by 7 years of age [5, 11]. Uncommonly, neurocognitive impairments are the presenting symptoms in the absence of clinical seizures but can be seen in up to 20% of cases [5]. Hyperactivity is also commonly noted as an accompanying behavioral symptom [13].

## 4. Electrophysiological Features

Most authors have agreed upon a few key criteria for defining ESES, including the activation of epileptiform discharges during sleep [9, 14], the presence of bilateral or sometimes unilateral continuous or near-continuous slow spike and waves [9, 14], and the occurrence of slow-spike-wave during a significant proportion of non-REM sleep [1, 2, 9, 14]. It was suggested that at the minimum, spike wave activity should occupy 85% of non-REM sleep in order to diagnose CSWS [1, 14]. The International League Against Epilepsy (ILAE) criteria do not specify a minimum percentage value for diagnosing ESES (Commission on Classification and Terminology of the International League Against Epilepsy, 1989). Discharges generally consist of a frequency between 1.5 to 3 hertz [2].

The spike-wave index (SWI) aims to quantify the frequency of spikes in the EEG record, thought of as the percentage of non-REM sleep occupied by spike waves [14]. Some have defined the SWI as the percentage of one-second bins occurring in non-REM sleep with greater than or equal to one spike and wave, relative to the total number of one-second bins occurring in non-REM sleep [15]. It is the highest in the early sleep cycles and decreases progressively over the course of a night [9]. Assessment of SWI may be more useful as a way to quantify evolution of the spike-wave activity within any given patient, as opposed to taking SWI as a fixed threshold for all patients [16].

Localization of electrographic findings tends to be frontotemporal or centrotemporal during both wakefulness and sleep, though during sleep, discharges tend to become much more widespread and frequent and commonly generalize [2].

The characteristic electrographic findings of stage 2 and 3 sleep are difficult to ascertain in the context of ESES, as spike-wave discharges disrupt the typical background findings. Electrographic REM sleep findings are more readily noticeable as the ESES activity is diminished, and rare spikes can be seen, similar to those seen during wakefulness [9, 14].

Interictal findings during wakefulness include focal or multifocal spikes, or slow waves, sometimes occurring in bursts. As neurocognitive regression occurs, the interictal abnormalities during wakefulness also become more prominent [2].

## 5. Neuroimaging Findings

**Figure 1:**
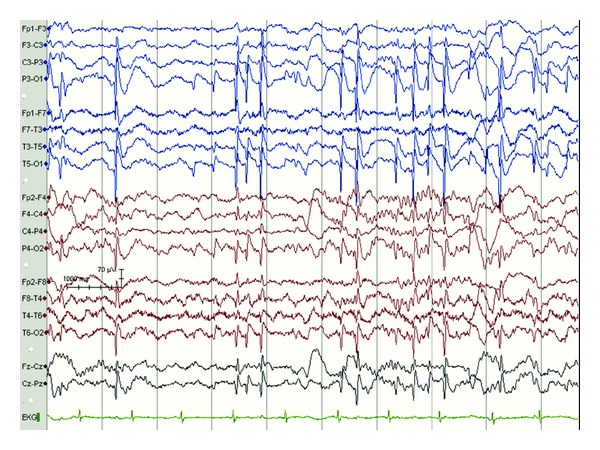
Asymmetric ESES with discharges seen more prominently over the left hemisphere, corresponding to unilateral structural injury; in this case, neonatal left thalamic stroke. Note the absence of spindles in left hemisphere further suggesting the possibility of thalamic injury. Recordings were obtained using standard international 10–20 electrode placement and a single electrocardiogram chest electrode. The recordings were obtained using a reference electrode and reformatted digitally into sequential bipolar and referential montages for review.

Some studies report abnormal neuroimaging findings in as many as 45–59% of cases of ESES [8, 17]. While there have been a variety of reported associated structural anomalies associated with CSWS, early developmental lesions such as perinatal vascular lesions are commonly reported, in as many as 21–78%; cortical malformations have been reported in up to 1/4 and abnormal myelination has been reported in another 10–15% [8, 15, 17]. Given this diversity, it is thought that CSWS represents an age-related response to any of multitude insults in susceptible children [8, 14]. These structural changes may result in asymmetric ESES on scalp EEG, seen ipsilateral to the injured side (Figure 1).

## 6. Pathophysiology

The mechanisms underlying development of the ESES pattern are complicated, though several hypotheses exist. One such explanation is that the corticothalamic network modulating oscillatory rhythms becomes pathologically hyperactivated, which may produce the ESES pattern seen on scalp EEG [2]. The thalamic circuit that gives rise to physiologic oscillations during sleep has been postulated to contribute to the discharges seen in ESES [18]. Reticular thalamic (RT) neurons provide inhibitory GABAergic inputs, while the dorsal thalamic nuclei's glutaminergic neurons provide excitation and the interplay between the two results in the oscillatory properties of the thalamus [18]. During wakefulness, the reticular activating system inhibits these oscillatory properties, and during non-REM sleep, these circuits are disinhibited [18]. It has been suggested that the change from physiologic oscillation to pathologic oscillation seen in ESES is related to the switch from GABA_A_-related inhibitory postsynaptic potentials to GABA_B_-mediated inhibitory postsynaptic potentials, which are associated with a longer latency and thus perhaps slowing frequencies seen [18]. It was shown that GABA_B_-receptor antagonists can eliminate the epileptiform activity occurring at 3 hertz *in vivo* [19]. In addition, it was shown that in a rat model of atypical absence epilepsy, GABA_B_-receptor antagonists treat the associated learning impairment [20]. Functional imaging studies evaluating the neuronal networks involved in CSWS have implicated the perisylvian regions, the prefrontal cortex, the cingulate gyrus, and the thalamus [21]. In CSWS, discharges activated and propagated bilaterally were seen [21].

## 7. Other Associated Conditions

There have been some conditions associated with the development of CSWS. Some reports have described CSWS associated with neurodegenerative disorders [22, 23]. In addition, there have been reports of associated congenital hydrocephalus and CSWS [24].

## 8. Outcomes

The natural history of seizures suggests that clinical seizures cease spontaneously, typically around puberty. This is seen regardless of associated etiological lesion and is seen in cases of fixed structural abnormalities as well as progressive degenerative conditions [23].

The natural history of the electrographic finding of ESES suggests that ESES ceases around age 11 years and thus has been described as a self-limited condition [11]. The EEG subsequently can continue to show focal discharges [9, 11].

Typically, improvement of clinical seizures and ESES is associated with cognitive improvement, though most patients continue to demonstrate some degree of impairment [2]. It has been proposed that frequent EEG spikes and ESES patterns are associated with poor neurocognitive outcome; thus, it has been presumed that improving the EEG appearance may positively impact cognitive outcome [25]. According to some reports, duration of ESES seems to be the main predictor of neurocognitive function [26, 27].

## 9. Traditional Treatment Strategies

Given the effect that seizure frequency and duration of ESES have on neurocognitive outcome, treatments have focused on minimizing clinical seizure frequency as well as targeting the ESES pattern. It seems that no one anticonvulsant is better than any other anticonvulsant [12]. Case series have described valproate, ethosuximide, and benzodiazepines as first-line therapies [2]. There have been case series reporting improving EEG abnormalities with high-dose diazepam given at nighttime [3, 25], though frequent relapses have also been reported, necessitating further rounds of treatment [25]. Polytherapy is frequently considered given the severity of this disorder [2]. Phenobarbital, phenytoin, and carbamazepine are typically avoided, as some reports have shown them to be either ineffective or even worsen the condition [26], although other reports describe no worsening and even improvement on these agents [6].

Treatments with medications other than traditional anticonvulsants, such as with steroids, adrenocorticotropic hormone (ACTH), or intravenous immunoglobulin (IVIg), have been used in patients who do not respond to traditional anticonvulsants [10, 17, 28]. One series of 44 children with ESES reported improvement in seizure frequency and neurocognitive functioning in up to 77% and a long-term remission rate of 45% of cases after prolonged treatment of 21 months of steroids [17]. Steroids on the order of high-dose corticosteroid pulse therapy or chronic oral steroid therapy at doses such as 1 mg/kg/day for 6 months have also been reported [29].

Another avenue for treatment is the ketogenic diet. The ketogenic diet is a high-fat, low-carbohydrate diet used in the treatment of intractable childhood epilepsy. It has been described as useful in children with a condition related to CSWS called the Landau-Kleffner syndrome [30]. A study evaluated the efficacy of the ketogenic diet on electroclinical characteristics and cognitive functioning in children with CSWS. The investigators described that in the study group of five children with CSWS refractory to conventional anti-epileptic drug therapy as well as steroid therapy and only one case responded with complete cessation of CSWS; another case demonstrated partial response, and three cases demonstrated no clear response. However, the authors acknowledge that the absence of a control group makes effectiveness on neurocognitive outcome impossible to conclude [31].

Surgical treatment is an option in those with focal lesions, even if the EEG pattern is generalized and includes therapies such as multiple subpial transections, focal resective surgery, and even hemispherectomy [2, 28]. Various case series have reported improvement in seizure burden and EEG pattern after surgical treatments [2], and there have been reports of improved neurocognitive outcomes in individuals following surgery for ESES [32], though large series of data regarding neurocognitive outcomes following surgery for ESES are lacking.

It seems that over half of all treated patients improve, but it is still unclear what effect these treatment strategies have on long-term outcomes, when considering the natural history of age-related improvement [2]. In addition, it is recommended that frequent neuropsychological assessments be undertaken, particularly for those children with a high SWI, who typically demonstrate more severe developmental disturbances [8, 13].

## 10. Newer Treatment Strategies

There have been some recent advances with experimental systems in potential treatments for those with ESES and CSWS, particularly those with drug-refractory conditions. One such system used transcranial DC stimulation (tDCS) in patients with drug-refractory CSWS. Electrical DC polarization of the brain has previously been demonstrated to change neuronal discharges in the cortices of animals [33, 34]. Continuous EEG monitoring was employed to evaluate electrographic activity throughout the procedure. Stimulation with 1 mA was demonstrated to be tolerated in both children with CSWS as well as controls, and two patients with CSWS demonstrated a large reduction in epileptiform discharges [35].

Further, our personal experience has demonstrated that pharmacologic coma with pentobarbital, titrated to a burst-suppression pattern on EEG, has shown some efficacy in clearing the scalp EEG findings of ESES. This method has been tried in those children whose ESES is refractory to all other traditional treatment approaches, including high-dose diazepam, intravenous pulse steroids, chronic daily steroids, and amantadine. While pentobarbital-induced suppression of EEG in the setting of ESES has demonstrated some efficacy in our experience, this is still at a case report level and ought to be studied further.

## 11. Related Conditions

CSWS is thought to lie on the more severe end of a clinical spectrum in which benign epilepsy of childhood with centrotemporal spikes (BECTS) and benign occipital epilepsy of childhood (BOEC) represent the milder end [2]. BECTS and BOEC are both age-related conditions characterized by focal spikes that are potentiated by sleep. Seizure semiology varies according to areas of cortex involved [2]. As these are relatively benign conditions, normal neurocognitive outcome has been classically considered as a defining feature. However, in more recent years, detailed neuropsychological testing has revealed that there may be subtle deficits in language, behavior, and learning in children with these disorders [36].

CSWS is also related to a condition known as acquired epileptic aphasia, or Landau-Kleffner syndrome (LKS). This is an acquired syndrome characterized by subacute and progressive language only dysfunction, that occurs in an age-related manner. Children with LKS present with an acquired auditory agnosia, or “word deafness” [28, 29], thought to be related to functional disturbance of posterior temporal language areas [29]. This disorder typically affects children between age 5 and 7 years. At the time of the loss of language, there can be associated behavioral dysfunction. There can be related clinical seizures, though these tend to be infrequent and easily controlled and typically are nocturnal focal motor seizures. The interictal EEG demonstrates bilateral posterior-temporal or parieto-occipital spikes, that are potentiated by and more broadly distributed during non-REM sleep, when they may resemble ESES. In this condition, similar to the natural history timeline of CSWS, the aphasia tends to improve around puberty [37]. In acquired epileptic aphasia, unlike in CSWS, there is only rarely an associated structural brain lesion [8, 15, 38]. While epilepsy in LKS tends to resolve with time, many affected children still manifest residual cognitive or language impairment, and the duration of ESES seems to be the major association with poor outcome [28].

Other less commonly associated conditions include the Lennox-Gastaut syndrome (LGS) or its variants. Unlike CSWS, in the Lennox-Gastaut syndrome, hyperexcitability during non-REM sleep is not a prominent feature. The electrical status epilepticus of LGS is not related to sleep and is also unrelated to age [39].

## 12. Conclusions

Continuous spike and wave during slow wave sleep (CSWS) is an epileptic encephalopathy that presents with neurocognitive regression and clinical seizures, and that demonstrates an electroencephalogram (EEG) pattern of electrical status epilepticus during sleep. While the pathophysiology is not completely understood, there are known genetic and developmental associations with this condition. Traditional anticonvulsants as well as hormonal therapies are used with mixed results. Only with improved understanding of the pathophysiology of this condition, will advances in treatment be achieved. The end goal remains to preserve neurocognitive development.
